# An early global role for Axin is required for correct patterning of the anterior-posterior axis in the sea urchin embryo

**DOI:** 10.1242/dev.191197

**Published:** 2021-03-31

**Authors:** Hongyan Sun, Chieh-fu Jeff Peng, Lingyu Wang, Honglin Feng, Athula H. Wikramanayake

**Affiliations:** Department of Biology, University of Miami, Coral Gables, FL 33146, USA

**Keywords:** Wnt signaling, Axin, Sea urchin, Anterior-posterior axis, Animal-vegetal axis, Endomesoderm

## Abstract

Activation of Wnt/β-catenin (cWnt) signaling at the future posterior end of early bilaterian embryos is a highly conserved mechanism for establishing the anterior-posterior (AP) axis. Moreover, inhibition of cWnt at the anterior end is required for development of anterior structures in many deuterostome taxa. This phenomenon, which occurs around the time of gastrulation, has been fairly well characterized, but the significance of intracellular inhibition of cWnt signaling in cleavage-stage deuterostome embryos for normal AP patterning is less well understood. To investigate this process in an invertebrate deuterostome, we defined Axin function in early sea urchin embryos. *Axin* is ubiquitously expressed at relatively high levels in early embryos and functional analysis revealed that Axin suppresses posterior cell fates in anterior blastomeres by blocking ectopic cWnt activation in these cells. Structure-function analysis of sea urchin Axin demonstrated that only its GSK-3β-binding domain is required for cWnt inhibition. These observations and results in other deuterostomes suggest that Axin plays a crucial conserved role in embryonic AP patterning by preventing cWnt activation in multipotent early blastomeres, thus protecting them from assuming ectopic cell fates.

## Abstract (precis)

**Summary:** Axin function is required in the early sea urchin embryo to regulate nuclear β-catenin levels and prevent ectopic cell fates in multipotent early blastomeres, and to ensure correct anterior-posterior axis patterning.

## INTRODUCTION

During embryonic development in most bilaterally symmetrical animals (Bilateria), the establishment of the anterior-posterior (AP) and dorsal-ventral (DV) axes are two early events that are crucial for normal embryogenesis. The coordinate system created by these axes provide the positional information required for the ordered morphogenesis and cell fate specification that leads to the formation of a complex three dimensional embryo (Niehrs, 2010). The AP axis is usually the first axis specified during early bilaterian embryogenesis and in many species its formation is strongly influenced by a polarity found in the unfertilized egg termed the animal-vegetal (AV) axis (Kume and Dan, 1968; Martindale, 2005; Martindale and Hejnol, 2009; Petersen and Reddien, 2009; Wilson, 1925). During early embryogenesis, maternal determinants that are asymmetrically localized at the vegetal pole of the egg are mobilized to activate the Wnt/β-catenin (cWnt) pathway in blastomeres that will later contribute to the future posterior end of the embryo (Loh et al., 2016; Martindale and Hejnol, 2009; Petersen and Reddien, 2009). Although observations from a number of deuterostome and protostome taxa indicate that cWnt signaling plays a crucial role in the specification and patterning of the AP axis during early embryogenesis in bilaterians (Martindale, 2005; Petersen and Reddien, 2009), the mechanisms that restrict the activation of cWnt to the posterior end of embryos remain poorly understood.

In addition to the role of cWnt signaling in specifying posterior cell fates in early embryos, there is now extensive evidence that correct AP axis patterning also requires the inhibition of cWnt signaling at the anterior end (Petersen and Reddien, 2009; Range, 2014; Yamaguchi, 2001). This process has been best described in vertebrate embryos, where anterior inhibition of cWnt signaling is typically mediated by secreted factors that directly bind the Wnt ligand or one of the Wnt co-receptors. Studies have shown that experimental downregulation of these inhibitors results in the loss of head structures, and, moreover, that ectopic expression of these factors can induce duplication of anterior structures (Bouwmeester et al., 1996; Cruciat and Niehrs, 2013; Ding et al., 2018; Glinka et al., 1998; Pera and De Robertis, 2000; Piccolo et al., 1999). Remarkably, work carried out in some invertebrate deuterostome embryos has shown that homologs of the vertebrate secreted cWnt inhibitors are also required for the correct patterning of the anterior end during embryogenesis in these taxa (Niehrs, 2010; Onai et al., 2012; Petersen and Reddien, 2009; Range and Wei, 2016). In summary, these data point to a conserved mechanism for AP axis patterning in deuterostome embryos where activation of cWnt signaling at the posterior end of the embryo specifies posterior cell fates and induces a posterior signaling center. In addition, an inhibition of cWnt signaling at the anterior pole is necessary for differentiation of anterior structures.

The observations made in deuterostome embryos indicate that extracellular cWnt inhibition at the anterior pole by secreted factors is a relatively late event during embryogenesis. However, recent work in the short germ band insect *Tribolium castaneum* has shown that there is an early inhibition of cWnt signaling at the future anterior end of these protostome embryos (Ansari et al., 2018; Fu et al., 2012; Prühs et al., 2017). Interestingly, in *T. castaneum* embryos, inhibition of cWnt signaling at the anterior end is mediated intracellularly by Axin, a crucial cytoplasmic component of the β-catenin destruction complex in the cWnt pathway (Ansari et al., 2018; Fu et al., 2012; Prühs et al., 2017). The β-catenin destruction complex is a highly conserved negative regulator of cWnt signaling in animals. In addition to Axin, the destruction complex is composed of three other major proteins: APC, the product of the adenomatous polyposis coli gene; Glycogen Synthase Kinase 3β (GSK-3β); and Casein Kinase 1α (CK1α). When the cWnt pathway is in an off state, cytosolic β-catenin binds to Axin and APC where it is phosphorylated by CK1α and GSK-3β, and targeted for degradation through the proteasome pathway (Aberle et al., 1997; Nusse and Clevers, 2017). During cWnt activation, a Dishevelled (Dvl)-mediated disruption of the destruction complex leads to the stabilization of β-catenin. Stabilized β-catenin then translocates into the nucleus where it binds to the LEF/TCF transcription factors and acts as a transcriptional co-activator to activate target genes (MacDonald et al., 2009; Nusse and Clevers, 2017). Mutations in Axin and/or APC that lead to impaired β-catenin regulation can lead to increased levels of cWnt signaling and result in disrupted development and numerous diseases, including cancer (Clevers and Nusse, 2012; MacDonald et al., 2009; Nusse and Clevers, 2017). In *T. castaneum, TcAxin* is expressed at the anterior end of unfertilized eggs and early embryos; strikingly, downregulation of Axin expression using RNAi resulted in the duplication of posterior structures at the anterior end of the embryo (Ansari et al., 2018; Fu et al., 2012; Prühs et al., 2017). The work carried out in *T. castaneum* is the first clear example of Axin-mediated downregulation of posterior fates at the anterior end, but earlier work carried out in *Xenopus* and mice demonstrated a role for Axin in preventing ectopic dorsal cells fates in ventral blastomeres in these vertebrate embryos (Kofron et al., 2001; Zeng et al., 1997). In summary, these observations suggest that active repression of the cWnt pathway in early embryos through an intracellular mechanism may be required for early axis formation in protostome and deuterostome embryos. However, many crucial details about the early role of Axin in modulating early axis patterning, particularly in deuterostomes, remain poorly understood.

In the sea urchin embryo, early AP axis specification and patterning is reminiscent of what is seen in other invertebrate bilaterian embryos. The nuclearization of β-catenin is seen in the four micromere cells at the vegetal pole as early as the 16-cell stage; by the 60-cell stage, β-catenin is seen in the nuclei of all vegetal cells that are specified as endomesoderm at this stage (Logan et al., 1999; Weitzel et al., 2004). Consistent with these observations, downregulation of early cWnt signaling leads to failure of endomesoderm specification and produces severely anteriorized embryos with ectopic expression of anterior neuroectodermal (ANE) markers throughout the embryo (Emily-Fenouil et al., 1998; Logan et al., 1999; Range et al., 2013; Wikramanayake et al., 1998). Moreover, ectopic activation of cWnt signaling in animal-half blastomeres produces embryos that are posteriorized with an expanded external gut and reduced ectoderm (Emily-Fenouil et al., 1998; Wikramanayake et al., 1998). The mechanisms that initially restrict cWnt signaling to the posterior end of 16-cell stage sea urchin embryos are not well understood but several studies have shown that it involves the local ‘activation’ of Dvl at the vegetal pole (Croce et al., 2011; Peng and Wikramanayake, 2013; Weitzel et al., 2004). There is also evidence that the initial activation of cWnt at the posterior end takes place in a Wnt ligand-independent manner (Cui et al., 2014; Logan et al., 1999), but this issue is not settled. During normal development, anterior blastomeres do not display nuclear β-catenin, but some experimental observations suggest that there is an active inhibition of cWnt at the anterior end during early embryogenesis. For example, when a β-catenin::GFP fusion protein was expressed in early embryos, its nuclearization was initially seen in all blastomeres. But β-catenin::GFP was then rapidly downregulated at the anterior end of the embryo and the fusion protein persisted in nuclei of posterior blastomeres (Weitzel et al., 2004). This study also demonstrated a role for GSK-3β in inhibiting β-catenin nuclearization in anterior blastomeres, suggesting a possible active role for the β-catenin destruction complex in this process (Weitzel et al., 2004).

In this study, we examined Axin function in the early sea urchin embryo to determine the precise role of this protein in regulating early patterning of the sea urchin embryo. We show that Axin functions in all blastomeres of the early sea urchin embryo to downregulate cWnt activation and that selective downregulation of Axin function in anterior blastomeres leads to induction of ectopic posterior cell fates in these cells. We also show that only the GSK-3β binding site on Axin is required for the cWnt inhibitory function of the protein, and our results indicate that the main function of Axin during cWnt regulation in the sea urchin is to bring GSK-3β to the destruction complex. We propose that Axin plays a crucial role in early AP axis patterning in embryos by blocking nuclearization of β-catenin in multipotent blastomeres to protect them from ectopic cell fate specification.

## RESULTS

In this study, we used the sea urchin species *S. purpuratus* and *L. variegatus.* All experiments except the experimental embryology studies were carried on both species. The species from which the data were generated is clearly indicated in each figure legend.

### Axin is maternally loaded and is dynamically expressed throughout early embryogenesis

**Fig. 1.**
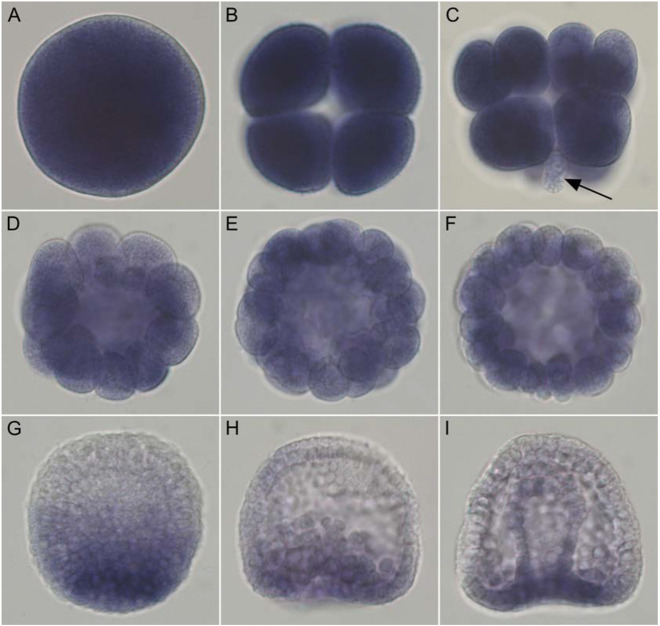
***SpAxin* mRNA is expressed maternally and dynamically throughout embryogenesis.** Images show the spatial distribution of *Axin* mRNA detected by whole-mount RNA *in situ* hybridization in *S. purpuratus* eggs and embryos. (A-F) *Axin* is ubiquitously expressed in the egg (A), and at the 8-cell stage (B), the 16-cell stage (C), 32-cell stage (D), 60-cell stage (E) and 120-cell stage (F). At the 16-cell stage, Axin displays lower expression in the vegetal micromeres (arrow in C). (G) At the hatching blastula stage, *Axin* mRNA is downregulated in anterior blastomeres. (H,I) By the early gastrula stage, *Axin* expression is restricted to the vegetal plate (H); by the late gastrula stage, expression is downregulated in the archenteron (I).

Previous studies have indicated that GSK-3β activity may inhibit ectopic β-catenin nuclearization at the anterior pole, presumably through its activity in the cWnt destruction complex (Emily-fenouil et al., 1998; Weitzel et al., 2004). As Axin is a major component of the cWnt destruction complex, we examined its spatial expression in eggs and early embryos to determine whether the expression was consistent with a role in inhibiting cWnt at the anterior pole (Fig. 1A-I, Fig. S2). In eggs and eight-cell stage embryos, *Axin* is ubiquitously expressed (Fig. 1A,B). *Axin* continues to be broadly expressed in 16-cell embryos but, interestingly, at this stage *Axin* mRNA expression appears to be downregulated in the four micromere cells that form at the vegetal pole (Fig. 1C). This observation raised the possibility that maternal *Axin* mRNA might be removed from the 16-cell stage micromeres in which the cWnt pathway is first activated or that *Axin* mRNA may be degraded in these cells (Fig. 1C). Between the 32- to 120-cell stages, the *Axin* message continues to be broadly expressed throughout the embryo (Fig. 1D-F). Previous studies have shown that nuclearization of β-catenin is initiated in 16-cell stage micromeres and β-catenin nuclearization expands to the veg2 tier, the cell tier immediately anterior to the micromeres, by the 60-cell stage (Logan et al., 1999). Activation of endomesodermal gene expression is initiated in the 16-cell stage micromeres; by the 60-cell stage, endomesoderm gene expression is seen robustly in the veg2 cell tier (Lhomond et al., 2012; Peter and Davidson, 2011; Sethi et al., 2012; Wikramanayake et al., 2004). Notably, at these stages, expression of *Axin* persists in anterior blastomeres. At around the hatching blastula stage, there is a downregulation of *Axin* in anterior blastomeres, but the gene continues to be expressed at relatively high levels in posterior blastomeres (Fig. 1G). At the early gastrula stage, *Axin* is seen at the vegetal plate and the invaginating gut (Fig. 1H), but by the mid/late gastrula stages *Axin* expression is downregulated in the archenteron, the skeletogenic primary mesenchyme and non-skeletogenic mesoderm cells, and it remains expressed at relatively high levels at the vegetal plate (Fig. 1I). Eggs and embryos incubated with the sense *Axin* probe showed no staining (Fig. S2). These data showed that spatial expression of *Axin* is consistent with a role for it in broadly inhibiting nuclear β-catenin in early blastomeres.

### Axin inhibits cWnt signaling in anterior blastomeres

**Fig. 2.**
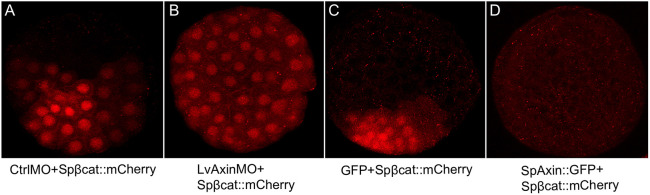
**Axin levels affect nuclearization of β-catenin in all blastomeres in sea urchin embryos.** (A) Embryos co-injected with control MO and *Spβ-catenin::mCherry* mRNA. β-Catenin::mCherry nuclearization is seen in posterior blastomeres, as expected. (B) Embryos co-injected with Axin MO and *Spβ-catenin::mCherry* mRNA. Nuclear β-catenin::mCherry is seen throughout the embryo. (C) Embryos co-injected with *GFP* and *Spβ-catenin::mCherry* mRNA. Nuclear β-catenin::mCherry was seen enriched at the posterior pole. (D) When *Axin* and *Spβ-catenin::mCherry* mRNA were co-injected, no nuclear β-catenin::mCherry was observed. For each case, we observed over 100 embryos and collected images from at least seven embryos. Experiments were carried out in *L. variegatus*.

In the early sea urchin embryo, *β-catenin* mRNA and protein are broadly distributed, including expression in the anterior blastomeres, where cWnt signaling is usually not activated (Logan et al., 1999; Miller and McClay, 1997). Immunostaining has shown that β-catenin protein is present in the cytoplasm, and at lateral cell-cell contacts and adherens junctions throughout the early embryo, consistent with its role in cell adhesion. But nuclearization of endogenous β-catenin protein is only seen in posterior cells (Logan et al., 1999; Miller and McClay, 1997). Because Axin is a major component of the β-catenin destruction complex, we predicted that if this complex was actively repressing cWnt signaling in anterior blastomeres then knockdown of Axin protein expression would result in ectopic nuclear β-catenin signaling in anterior cells. To test this idea, we used anti-Axin morpholinos to knock down Axin in embryos expressing *β-catenin::mCherry* mRNA and examined developing embryos for nuclearization of the fusion protein. This analysis showed that while β-catenin::mCherry nuclearization was restricted to the posterior end of control MO-injected embryos (Fig. 2A), β-catenin::mCherry was nuclearized in all cells in Axin-knockdown embryos (Fig. 2B). As we do not have a working anti-Axin antibody, we were unable to detect endogenous Axin protein expression, but these results are consistent with its efficient knockdown at a time when *Axin* mRNA is present in all blastomeres. From this result, we conclude that Axin protein suppresses cWnt activation in blastomeres at the future anterior pole of the early sea urchin embryo.

**Fig. 3.**
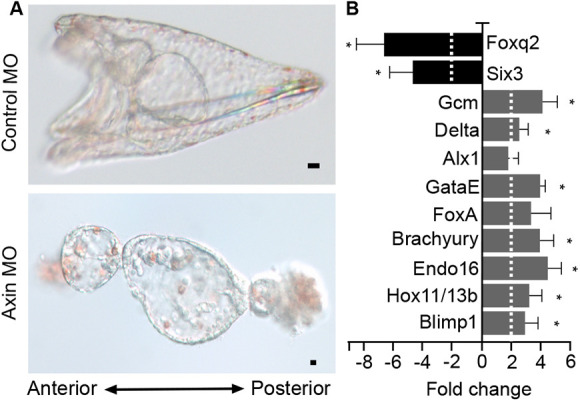
**Knockdown of Axin posteriorizes sea urchin embryos.** (A) Top panel shows a control pluteus-stage larva and the bottom panel shows an Axin-knockdown embryo at the same stage. (B) Gene expression in control and Axin-knockdown embryos at the hatching blastula stage analyzed using qPCR. The bar graph shows the fold change in expression of each gene between Axin MO- and control MO-injected embryos. *Blimp1*, *Hox11/13b*, *Endo16*, *Brachyury*, *FoxA*, *GataE*, *Alx1*, *Delta* and *GCM* are endomesoderm gene markers; *Foxq2* and *Six3* are ANE markers. qPCR experiments were replicated three times with three technical replicates for each experiment. Dashed line indicates a two-fold change. Data are mean±s.e.m. *P<0.05. Scale bars: 10 µm. Experiments were carried out in *S. purpuratus*.

Previous studies have shown that the ectopic activation of cWnt throughout the sea urchin embryo leads to a ‘vegetalization’ or posteriorization of embryos (Emily-fenouil et al., 1998; Logan et al., 1999; Wikramanayake et al., 1998). We followed control- or Axin-morpholino-injected embryos, and noted that they developed at similar rates through the hatching blastula stage with no obvious morphological differences (Fig. S3A,A′,B,B′). When control embryos were at the gastrula stage (Fig. S3C), the Axin knockdown embryos showed an exogastrulated phenotype (Fig. S3C′). When control embryos were at the pluteus stage (Fig. 3A, Fig. S3D), Axin-knockdown embryos showed the striking posteriorized phenotype seen only when cWnt signaling is ectopically activated (Fig. 3A, Fig. S3D′) (Emily-fenouil et al., 1998; Logan et al., 1999; Wikramanayake et al., 1998). This posteriorized phenotype indicated that Axin morphants underwent excess endomesoderm development with a concomitant reduction of ectoderm derivatives. To verify this, we used qPCR to analyze gene expression in control- and Axin-knockdown embryos at the hatching blastula stage. As predicted from the phenotype, qPCR analysis showed that several endomesoderm specific genes were upregulated in Axin-knockdown embryos compared with controls, while two ANE marker genes, *Six3* and *Foxq2*, were significantly downregulated (Fig. 3B). Importantly, Axin-knockdown using species-specific morpholinos produced the same phenotype in *S. purpuratus* and *L. variegatus*, indicating the specificity of the knockdown (Fig. 3, Fig. S3). Additional controls for morpholino specificity are described later.

**Fig. 4.**
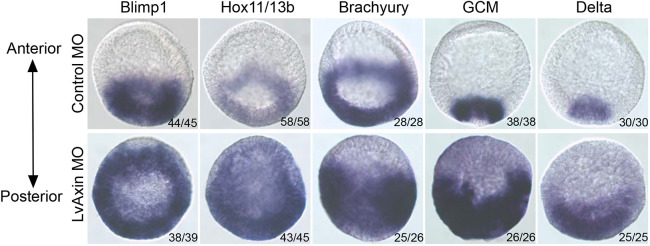
**Axin knockdown leads to ectopic expression of endomesoderm genes in anterior blastomeres.** Expression of selected endomesodermal gene markers in control (top) and Axin-knockdown (bottom) *L. variegatus* embryos was detected using whole-mount *in situ* hybridization. The number of embryos showing the expression pattern shown in the figures is indicated.

To determine whether the upregulation of endomesoderm gene expression seen in the qPCR analysis was due to ectopic endomesoderm gene expression at the anterior pole, we carried out whole-mount *in situ* hybridization. This analysis showed that the endomesodermal genes assayed were ectopically expressed in anterior blastomeres (Fig. 4). Interestingly, although all markers tested showed ectopic expression in anterior blastomeres, none of the markers was expressed at the most animal-pole domain of blastula stage embryos, suggesting that cells at this region of the embryo were not responsive or were less responsive to cWnt signaling. This is not completely unexpected as previous studies have shown that the expression of Wnt inhibitors at the ANE protects this domain from a posterior Wnt signaling cascade (Range et al., 2013). However, the restricted expression of Wnt inhibitors at the ANE normally occurs after the hatching blastula stage (Range, 2014). Hence, it is possible that there are additional mechanisms to prevent cWnt activation in the most anterior blastomeres in early embryos, but further studies are needed to test this idea.

**Fig. 5.**
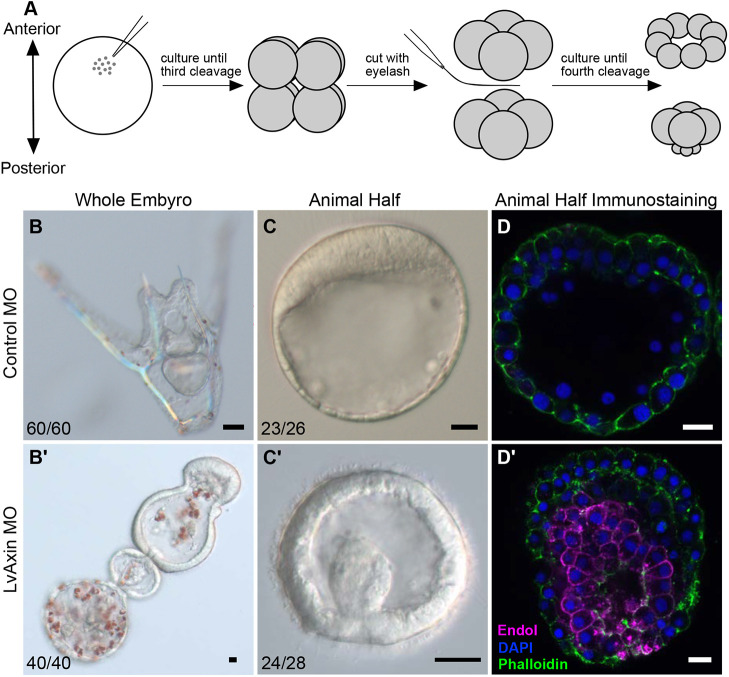
**Axin knockdown induces endoderm formation in isolated anterior blastomeres.** (A) Protocol for isolating animal halves following morpholino injection. (B) Control pluteus larva. (B′) Axin-knockdown embryo at same stage as in B. (C,C′) Embryoids from control animal half (C) and Axin-knockdown animal half (C′). (D,D′) Endo1 expression in isolated animal halves injected with control or Axin morpholinos. Magenta, Endo1; blue, DAPI; green, phalloidin. Scale bars: 10 µm. Experiments were carried out in *L. variegatus.*

The above results strongly indicated that Axin directly suppresses endomesoderm formation in anterior blastomeres by downregulating cWnt. However, it is known that cWnt-dependent signals from posterior cells starting around the 16-cell stage can influence the development of animal pole-derived blastomeres (Range, 2014; Wikramanayake and Klein, 1997; Wikramanayake et al., 1998). Hence, it was formally possible that downregulation of Axin in posterior blastomeres could enhance cWnt signaling in these cells and indirectly lead to nuclearization of β-catenin in anterior blastomeres. To directly test whether Axin function in anterior blastomeres was cell-autonomously required to suppress cWnt signaling, we examined endoderm formation in animal halves derived from eight-cell stage embryos injected with control or Axin morpholinos (Fig. 5A). As shown earlier, control embryos developed into pluteus larvae, while Axin-knockdown embryos became posteriorized (Fig. 5B,B′). As previously described, control animal halves made from eight-cell stage embryos developed into morphologically distinct polarized embryoids that do not form any endoderm or mesoderm (Fig. 5C; Wikramanayake et al., 1995, 1998). In contrast, Axin-deficient animal halves formed endoderm and gastrulated (Fig. 5C′). Endo1 expression in embryoids collected from Axin-knockdown embryos confirmed the development of endoderm, supporting the hypothesis that Axin cell-autonomously suppresses endodermal cell fate in anterior blastomeres (Fig. 5D,D′).

### Axin regulates cWnt signaling and endomesoderm specification at the posterior pole

In an earlier study, Range et al. (2013) showed that overexpression of Axin in the sea urchin embryo produced the stereotypical anteriorized phenotype (Emily-fenouil et al., 1998; Logan et al., 1999; Wikramanayake et al., 1998). Strikingly, analysis of ANE markers in these embryos showed that they were broadly expressed in most cells (Range et al., 2013). As Axin is a strong negative regulator of cWnt signaling, this result was consistent with evidence that cWnt pathway-dependent signals from posterior cells restrict the ANE domain to the anterior pole of the embryo (Martínez-Bartolomé and Range, 2019; Range et al., 2013; Range, 2014; 2018). However, the molecular mechanisms by which Axin overexpression expands ANE gene expression remain undefined. We therefore examined cWnt-dependent processes in Axin-overexpressing embryos. To test whether Axin regulates β-catenin nuclearization, we co-expressed β-catenin::mCherry with either GFP or SpAxin::GFP by mRNA injection. Imaging of 28- to 60-cell stage control embryos showed restricted nuclearization of β-catenin::mCherry at one pole of the embryo but, in contrast, embryos co-expressing Axin::GFP and β-catenin::mCherry were devoid of nuclear β-catenin::mCherry (Fig. 2C,D). These results provide evidence that Axin negatively regulates cWnt activation in early sea urchin embryos by inhibiting nuclear β-catenin – most likely by affecting its stability within the destruction complex.

**Fig. 6.**
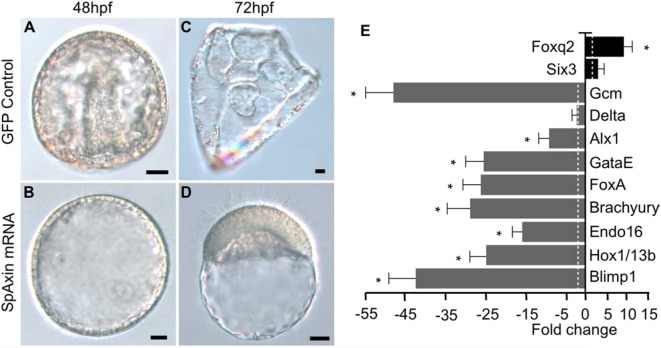
**Axin overexpression anteriorizes sea urchin embryos.** (A,C) Control embryos. (B,D) Axin-overexpressing embryos. (E) Gene expression in Axin-overexpressing embryos. The expression of selected gene markers in *Axin* and *GFP* mRNA-injected embryos at the hatching blastula stage was compared using qPCR. The bar graph shows the fold change of each gene between *Axin* mRNA-injected and *GFP* mRNA-injected control embryos. *Blimp1*, *Hox11/13b*, *Endo16*, *Brachyury*, *FoxA*, *GataE*, *Alx1*, *Delta* and *GCM* are endomesoderm gene markers; *Foxq2* and *Six3* are ANE markers. qPCR experiments were replicated with three separate batches of embryos with three technical replicates in each experiment. Dashed line indicates a twofold change in gene expression. Scale bars: 10 µm. For each experiment, 200-300 embryos were injected for each construct, and more than 95% of embryos had the same morphology as shown in the figures. *P<0.05. Experiments were carried out in *S. purpuratus*.

To determine the effect of Axin overexpression on endomesoderm specification, we observed the morphology of these embryos and assayed them for endomesoderm and ANE gene expression. Embryos injected with *Axin::GFP* mRNA developed at similar rates to control *GFP* mRNA-injected embryos (not shown). However, when control embryos were at the gastrula stage, Axin-overexpressing embryos remained in a blastula-like morphology (Fig. 6A,B). When control embryos were at the pluteus stage, Axin-overexpressing embryos displayed a phenotype consistent with the anteriorized phenotype generated by modulation of other intracellular cWnt components such as β-catenin, GSK-3β and LEF/TCF (Fig. 6C,D; Emily-Fenouil et al., 1998; Logan et al., 1999; Vonica et al., 2000; Wikramanayake et al., 1998). Analysis of gene expression in Axin-overexpressing embryos showed a downregulation of endomesoderm markers and an upregulation of ANE markers (Fig. 6E). These results confirmed the previous observations of Range et al. (2013) on the effects of Axin overexpression on ANE gene expression. In addition, these experiments demonstrated that elevation of Axin levels inhibited nuclearization of β-catenin, one of the earliest steps in endomesoderm specification in the sea urchin embryo.

### Structure-function analysis reveals that only the GSK-3β-binding domain of Axin is required for its activity in the cWnt pathway

**Fig. 7.**
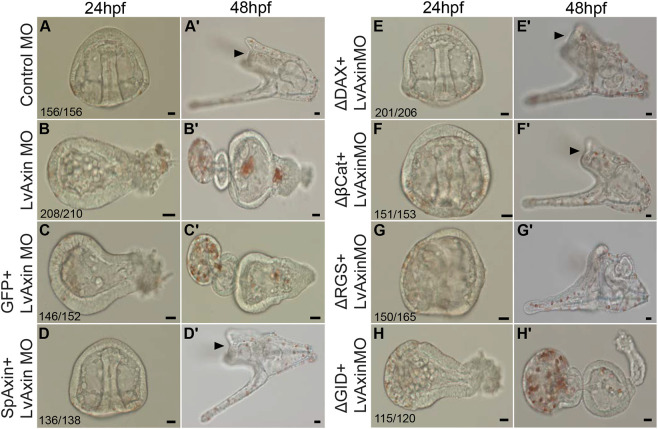
**The GSK-3β-binding domain of Axin is required for rescue of Axin-knockdown embryos.** (A,A′) Control embryos. (B,B′) Axin-knockdown embryos. (C,C′) Axin MO and *GFP* mRNA co-injected embryos. These embryos are posteriorized similar to those injected with Axin MO only (B,B′). (D,D′) Axin MO and *Axin* mRNA co-injected embryos. These embryos are indistinguishable from control MO-injected embryos (A,A′). (E,E′) Axin MO and *Axin ΔDAX* mRNA, and (F,F′) Axin MO and *Axin Δβcat* mRNA co-injected embryos. The Axin-knockdown phenotype is rescued in these embryos. (G,G′) Axin MO and *Axin ΔRGS* mRNA co-injected embryos. The Axin-knockdown phenotype is rescued in these embryos but when controls are at the pluteus stage, these embryos consistently have defects in the formation of the oral hood. Compare G′ with A′,D′,E′,F′. (H,H′) Axin MO and *Axin ΔGID* mRNA co-injected embryos. The Axin-knockdown phenotype is not rescued in these embryos. Each experiment was repeated three times. The numbers shown in each panel represent the number of embryos showing the phenotype shown in the panel out of the total number counted in an experiment. Scale bars: 10 µm. Arrowheads indicate the oral hood. Experiments were carried out in *L. variegatus*.

As a critical scaffolding protein in the cWnt destruction complex Axin interacts with several other proteins to regulate β-catenin stability (Luo and Lin, 2004; Schaefer and Peifer, 2019). The Axin proteins in bilaterians have well-characterized domains that mediate its interactions with APC (RGS), GSK-3β (GID), and β-catenin (βCat), and less well defined binding sites that mediate interactions with CK1α and Protein Phosphatase 2A (PP2A) (Luo and Lin, 2004; Schaefer and Peifer, 2019; Stamos and Weis, 2013). In addition, Axin has a domain that allows it to interact with Dvl (DAX) when the cWnt pathway is activated. Interestingly, the APC protein has multiple β-catenin-interacting domains that are distinct from the β-catenin binding domain in Axin. Despite extensive studies the significance of the β-catenin binding domain of Axin in regulating β-catenin stability is still unclear (Schaefer and Peifer, 2019; Stamos and Weis, 2013). To determine the importance of the four main domains on Axin in regulating β-catenin stability in sea urchins, we deleted each domain from SpAxin and tested the ability of each deletion-construct to rescue the Axin MO-mediated posteriorized phenotype (Fig. 7, Fig. S4). As expected, control MO-injected embryos developed normally through the gastrula and pluteus larvae stages (Fig. 7A,A′) and Axin MO-injected embryos were severely posteriorized (Fig. 7B,B′). When Axin MO was co-injected with *GFP* mRNA the embryos remained posteriorized, while the Axin-knockdown phenotype was completely rescued by *SpAxin::GFP* mRNA co-injection (Fig. 7C,C′,D,D′). Similar to rescue of Axin-knockdown embryos by full-length SpAxin, the SpAxin ΔDAX- and ΔβCat-binding domain deletion-constructs were able to rescue Axin-knockdown embryos (Fig. 7E-F′). However, we noted that although the Axin ΔRGS was able to rescue the posteriorized phenotype, the pluteus larvae did not develop a complete oral hood normally present in larvae (Fig. 7G,G′). Strikingly, in contrast to the complete or partial rescue activity of the SpAxin ΔDAX-, ΔβCat- and ΔRGS-domain deletion constructs, the SpAxin ΔGID was unable to rescue Axin-knockdown embryos (Fig. 7H,H′). Overall, these results showed that the full-length SpAxin was able to rescue Axin-knockdown mediated posteriorized phenotype, and, moreover, that the GID domain is the only domain required for this rescue activity.

**Fig. 8.**
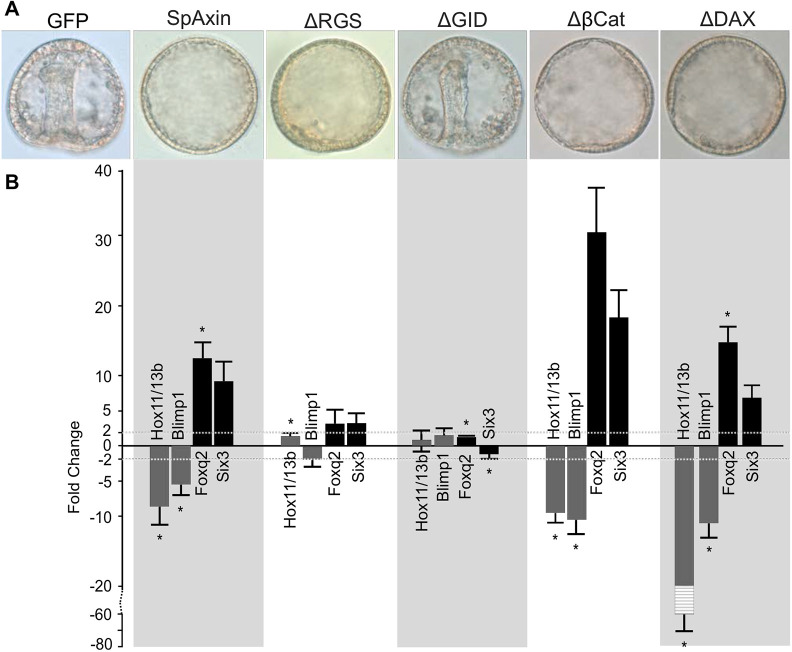
**Structure-function analysis of Axin function in regulating anterior-posterior axis patterning.** (A) The morphology of gastrula stage sea urchin embryos overexpressing full-length Axin and each of the single domain deletion constructs. Overexpression of full-length Axin, Axin Δβcat and Axin ΔDIX constructs by mRNA injection into zygotes blocked gastrulation, downregulated endomesoderm gene expression and increased ANE gene expression. Overexpression of Axin ΔRGS led to embryos that did not gastrulate, but qPCR analysis showed relatively normal gene expression, indicating that they were not anteriorized. Overexpression of Axin ΔGID had no effect on embryo development, indicating that this domain is required for the anteriorizing effect on sea urchin embryos when overexpressed. (B) Gene expression in hatching blastula stage sea urchin embryos overexpressing full-length Axin and each of the single domain deletion constructs. The *y*-axis shows fold change in gene expression between embryos expressing Axin constructs and embryos expressing GFP. Dashed line indicates a twofold change. Data are mean±s.e.m. *P<0.05. A scale break is used on the *y*-axis (−20 to −80) to adjust for the level of Hox11/13b fold change in the Axin ΔDIX-overexpressing embryos. The reason for this steep downregulation of Hox11/13b expression in Axin ΔDIX-overexpressing embryos is not known. Experiments were carried out in *S. purpuratus*.

The results described above indicated that the GID domain is the most important domain within SpAxin for regulating β-catenin stability in the cWnt pathway in sea urchin embryos. To further test this hypothesis, we overexpressed the SpAxin deletion constructs by mRNA injection into zygotes and assayed the ability of each to anteriorize sea urchin embryos. As previously shown, when control GFP-overexpressing embryos were at the gastrula stage, full-length SpAxin::GFP-overexpressing embryos were severely anteriorized (Fig. 8A,B). Similarly, overexpression of the Axin ΔβCat- and Axin ΔDAX-deletion constructs inhibited endomesoderm gene expression, blocked gastrulation and elevated ANE gene expression, consistent with the typical anteriorized phenotype in sea urchin embryos (Fig. 8A,B). In our replacement analysis, Axin ΔRGS was able to almost completely rescue the phenotype induced by knockdown of endogenous Axin (Fig. 7G,G′). Consistent with its functionality, overexpression of Axin ΔRGS acted dominantly to inhibit gastrulation and the resulting morphology superficially resembled anteriorization (Fig. 8A). Surprisingly, however, these embryos did not show any inhibition of endomesoderm gene expression or a significant elevation of ANE gene expression, indicating that they were not anteriorized (Fig. 8B). The intact AP polarity indicated that this construct likely did not affect β-catenin stability in the early embryo. However, inhibition of gastrulation resulting from overexpression of this construct indicated that deletion of the Axin RGS domain may specifically affect sea urchin development by possibly interfering with a β-catenin-independent pathway. The results from overexpression of the Axin ΔRGS construct (Fig. 8A,B) and from the rescue assay described earlier (Fig. 7G,G′) suggest that the Axin RGS domain may mediate an Axin function independent of the cWnt pathway, but this has to be more carefully examined in future studies.

**Fig. 9.**
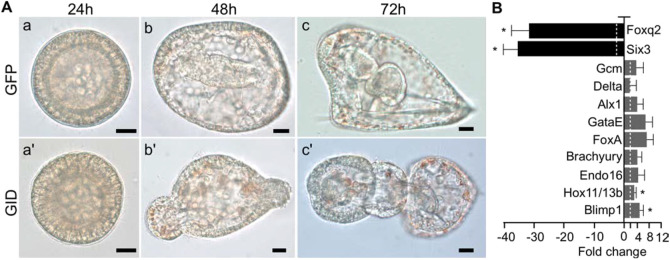
**Overexpression of the GID domain of Axin posteriorizes sea urchin embryos.** (Aa-c) Control embryos. (Aa′-c′) *Axin GID::GFP* mRNA-injected embryos. By the time the control animals are at the gastrula and pluteus stages, Axin GID::GFP-expressing animals have a severely posteriorized phenotype. Scale bars: 10 µm. (B) Gene expression in Axin GID::GFP-overexpressing hatching blastula stage embryos was assayed using qPCR. qPCR experiments were replicated three times with three technical replicates for each experiment. Dashed line indicates a twofold change. Data are mean±s.e.m. *P<0.05. Experiments were carried out in *S. purpuratus*.

An intriguing observation from the Axin-knockdown rescue experiments was that the SpAxin GID domain was required for rescue of the posteriorized phenotype (Fig. 7H,H′). To test whether the GID domain was also required for the anteriorizing activity of Axin, we overexpressed SpAxin ΔGID by mRNA injection into zygotes (Fig. 8A). Strikingly, embryos overexpressing SpAxin ΔGID were morphologically indistinguishable from control embryos and displayed relatively normal gene expression (Fig. 8A,B). These observations pointed to the conserved importance of the Axin GSK-3β-binding domain in mediating the function of Axin in the destruction complex. Consistent with this notion, previous work showed that overexpression of the Axin GID construct in the *Xenopus* embryo strongly inhibited GSK-3β and ectopically activated cWnt signaling, leading to duplicated dorsal axial structures (Hedgepeth et al., 1999). To determine whether the Axin GID domain alone had an effect on sea urchin embryos, we overexpressed *Xenopus* Axin GID::GFP and observed the embryos over time. At 24 h post-fertilization, control GFP- and GID-expressing embryos appeared to be morphologically similar (Fig. 9Aa,a′). When control embryos reached the gastrula and pluteus stages, the Axin GID::GFP expressing embryos displayed a severely posteriorized phenotype similar to that observed with Axin knockdown (Fig. 9Ab,b′,c,c′, Fig. S5). Furthermore, analysis of endomesodermal gene expression strongly supported ectopic activation of cWnt signaling in these embryos (Fig. 9B). Taken together, these results indicate that the GSK-3β-binding domain in sea urchin Axin is the only domain required for mediating its cWnt inhibitory function in the sea urchin embryo.

## DISCUSSION

The results of our studies revealed that Axin plays a crucial role in AP axis formation by preventing the ectopic activation of cWnt at the anterior end in early sea urchin embryos, thereby suppressing posterior cell fates in anterior blastomeres. These results and observations in other deuterostomes suggest that Axin – in its role in the destruction complex – may have a conserved role in globally preventing cWnt activation in rapidly dividing early embryos, thus protecting blastomeres from assuming ectopic cell fates. Additionally, structure-function analysis of sea urchin Axin indicated that it has domains that have conserved and divergent functions with the GSK-3β-binding region being required for the cWnt inhibitory activity of the protein.

### Axin inhibits cWnt signaling in anterior blastomeres during cleavage stages in the early sea urchin embryo

During early development in the sea urchin *Axin* mRNA is broadly expressed, including in blastomeres at the anterior pole. Knockdown of Axin showed that the protein is required for preventing nuclearization of β-catenin in anterior blastomeres thus blocking ectopic induction of endomesodermal cell fates. Past work has shown that the nuclearization of β-catenin in early sea urchin embryos is cell-autonomous and it most likely occurs independent of secreted Wnt ligands (Cui et al., 2014; Logan et al., 1999). Therefore, it is likely that during the cleavage stages, Axin cell-autonomously blocks β-catenin nuclearization in anterior blastomeres and possibly in all blastomeres prior to the 16-cell stage. This is the expected function for the destruction complex in cells, but we propose that in early embryos where blastomeres have broad developmental potential and rapid cleavage cycles, the activity of the destruction complex is particularly critical for tightly regulating cytoplasmic β-catenin levels. There is evidence from tissue culture studies that cWnt signaling fluctuates with the cell cycle with peak levels of signaling at the M phase (Niehrs and Acebron, 2012). This signaling correlates with fluctuating β-catenin levels in the cytoplasm that is independent of the β-catenin pool associated with cadherins and the adhesion complex (Olmeda et al., 2003). To the best of our knowledge, there are no published studies that document the cycling of cWnt signaling activity with the cell cycle in cleavage stage embryos. However, if fluctuations of cWnt signaling similar to those documented in tissue culture cells occur in early cleavage stage embryos, a tight regulation of the protein may be crucial. Early blastomeres generally have a broad potency and experimental manipulation can respecify blastomeres from their normal fates (Horstadius, 1973; Slack, 1991). Hence, ectopic activation of a powerful signaling pathway, such as cWnt in early blastomeres, could easily disrupt normal patterning in the early embryo, as has been shown in many experimental studies in sea urchins (Emily-fenouil et al., 1998; Vonica et al., 2000; Wikramanayake et al., 1998). In the sea urchin embryo, the potential of anterior blastomeres to respond to activators of cWnt is lost by the late blastula, suggesting that the fates of blastomeres have been determined by this stage (Livingston and Wilt, 1992; Nocente-McGarth et al., 1991; Wikramanayake and Klein, 1997). Interestingly, this corresponds to the time when Axin expression is downregulated in anterior blastomeres (see Fig. 3). The downregulation of *Axin* in anterior blastomeres at the late blastula stage may indicate that high levels of Axin and the destruction complex are not needed in these blastomeres for β-catenin regulation in the cytoplasm once cell divisions have slowed down and cell fates have been determined.

Work carried out in other taxa suggests that the requirement for Axin and the destruction complex to protect early blastomeres from activation of cWnt signaling in cleavage stage embryos may be broadly conserved. For example, our results are similar to observations previously reported in *T. castaneum* where *TcAxin* is expressed at the anterior end of unfertilized eggs and early embryos (Ansari et al., 2018; Fu et al., 2012; Prühs et al., 2017). Similar to what we report for sea urchins, downregulation of Axin in *T. castaneum* led to the duplication of posterior structures at the anterior end (Ansari et al., 2018; Fu et al., 2012; Prühs et al., 2017). However, in the case of *T. castaneum, Axin* is tightly localized to the anterior end of the egg and early embryo through the activity of Tc Germ cell-less (Ansari et al., 2018). But in mice, frogs and zebrafish, Axin is broadly distributed in the egg and early embryo, as seen in the sea urchin (Fig. 1), and downregulation of Axin in these vertebrates resulted in duplicated dorsal axial structures due to ectopic activation of cWnt signaling (Heisenberg et al., 2001; Kofron et al., 2001; Zeng et al., 1997). In the invertebrate deuterostome *Amphioxus*, the expression of *Axin* is very similar to what we have reported in sea urchins, with ubiquitous early expression in the egg and early embryo, and a ring of expression at the base of the archenteron at the gastrula stage (Onai, 2019). No functional studies have been carried out on Axin in this species but it would be of interest to determine whether Axin functions to suppress cWnt signaling in anterior blastomeres in early *Amphioxus* embryos and embryos of other species that may have global expression of Axin during early development. In summary, these observations in a number of taxa suggest that global activity of the destruction complex in early embryos may be an evolutionary adaptation to tightly regulate cWnt signaling in early embryos to protect multi-potent early blastomeres from β-catenin-induced cell fate changes.

### Structure-function analysis reveals conserved and divergent roles for Axin in the sea urchin embryo

In the sea urchin embryo, activation of cWnt signaling in posterior cells specifies endomesoderm but, in addition, cWnt signaling also activates a posterior signaling center that regulates patterning along the entire AP axis (Martínez-Bartolomé and Range, 2019; Range, 2014; Range et al., 2013; Wikramanayake et al., 1998). This global influence of cWnt signaling can be seen in experimental perturbations where blocking the nuclearization of β-catenin in cleavage stage embryos induces a unique phenotype that is now well characterized as an extreme anteriorization of embryos (Logan et al., 1999; Range et al., 2013; Wikramanayake et al., 1998). Mechanistically, this anteriorization is due to the disruption of the cWnt-initiated signaling cascade from posterior cells that progressively restricts ANE gene expression to the apical plate at the anterior end of the embryo. In our current work, we have extended the observations of Range et al. (2013) to show that upregulation of Axin levels by mRNA injection results in inhibition of nuclear β-catenin in posterior cells and inhibition of endomesoderm gene expression. How Axin overexpression leads to inhibition of nuclear of β-catenin at the posterior pole is not clear. As an intact Axin/APC complex is required for destruction complex function, it is possible that APC is not limiting and hence increased levels of Axin would result in increased levels of the Axin/APC scaffold that captures and targets more β-catenin for degradation. Alternatively, it is possible that overexpressed Axin competes with the endogenous intracellular signaling mechanism that downregulates the destruction complex in posterior cells. This would keep the endogenous destruction complex functional in posterior cells, leading to inhibition of nuclear of β-catenin. Additional experiments are required to distinguish between these possibilities.

Many *in vitro* studies carried out in cultured cells and *in vivo* studies carried out primarily in vertebrates and *Drosophila* have defined the domains that allow Axin to interact with crucial cytoplasmic components involved in regulating cWnt signaling, including APC, GSK-3β, β-catenin and Dvl (Kikuchi, 1999; Luo and Lin, 2004; Schaefer and Peifer, 2019; Song et al., 2014; Stamos and Weis, 2013; Tacchelly-Benites et al., 2013). Studies have shown that Axin interacts with APC via the RGS domain and to a lesser extent through other regions of the protein (Luo and Lin, 2004; Schaefer and Peifer, 2019; Stamos and Weis, 2013). Work carried in vertebrates has shown that overexpression of an Axin construct lacking the RGS domain induces ectopic dorsal structures in ventral blastomeres by acting as a dominant negative (Fagotto et al., 1999; Hedgepeth et al., 1999; Zeng et al., 1997). In our studies, overexpression of the Axin ΔRGS construct failed to posteriorize embryos in the same way that a dominant negative to endogenous Axin would do in sea urchin embryos. Analysis of gene expression in Axin ΔRGS-overexpressing embryos showed relatively normal endomesoderm gene expression. However, Axin ΔRGS-overexpressing sea urchin embryos failed to extend an archenteron, suggesting a defect in morphogenesis. Whole-mount *in situ* hybridization analysis showed that *Axin* is expressed at the vegetal plate in embryos undergoing primary invagination (Fig. 1H). Axin expression is downregulated in the extended archenteron but it remains expressed as a ring at the vegetal plate of gastrula stage embryos (Fig. 1I). It is possible that Axin regulates a morphogenetic event such as apical constriction, primary invagination, or convergence and extension of the gut; the Axin ΔRGS construct somehow antagonizes this process. These processes, however, are not thought to typically involve cWnt signaling (Butler and Wallingford, 2017; Martin and Goldstein, 2014; Sawyer et al., 2010). In a study carried out in *Xenopus*, Schneider et al. (2012) showed that overexpression of an Axin mutant – in which the putative small GTPase-interacting domain on the RGS domain was mutated to make it non-functional as a GTPase-activating protein (GAP) – could not rescue the anterior brain deficits in Axin-knockdown embryos. These authors proposed that, during AP patterning of the *Xenopus* central nervous system, an Axin-RGS domain-mediated function at the level of small G-protein interaction may be required to attenuate cWnt signaling. It is possible that Axin plays a similar role in modulating cWnt signaling during the initiation of gastrulation in the sea urchin embryo. Alternatively, because it is known that the primary invagination and extension of the archenteron in the sea urchin embryo requires cell shape changes involving small G-protein signaling, it is possible that Axin has a role in morphogenesis of the gut through its activity as a GAP in a β-catenin-independent pathway (Beane et al., 2006; Croce et al., 2006; Wessel and Wikramanayake, 1999). This would not be an unprecedented role for Axin because other studies have implicated this protein as being involved in Wnt/β-catenin-independent signaling pathways (Luo and Lin, 2004).

In our studies, we show that deletion of Axin GID completely abolishes its ability to rescue the Axin-knockdown phenotype and to anteriorize sea urchin embryos when overexpressed (Figs 7H,H′ and 8A). The importance of the Axin GID for efficient destruction complex function has been established in many studies. For example, overexpression of Axin constructs lacking the GID in *Xenopus* failed to ventralize embryos, while a *Drosophila* Axin ΔGID protein expressed at near endogenous levels was unable to rescue embryos. Presumably, the loss of activity of these proteins was due to their inability to regulate the β-catenin destruction complex (Fagotto et al., 1999; Hedgepeth et al., 1999; Itoh et al., 1998; Kremer et al., 2010; Peterson-Nedry et al., 2008). We showed that overexpression of Axin GID alone produced a phenotype reminiscent of those produced by downregulation of GSK-3β activity or overexpression of β-catenin (Emily-Fenouil et al., 1998; Wikramanayake et al., 1998). It is not clear how overexpression of GID posteriorizes sea urchin embryos but, in *Xenopus* embryos, the GID construct can strongly inhibit GSK-3β enzyme activity (Hedgepeth et al., 1999). Interestingly, these authors showed that the GID can inhibit GSK-3β activity *in vivo* but not *in vitro*, and they suggested that additional factors are required for GID to inhibit GSK-3β activity *in vivo* (Hedgepeth et al., 1999). If similar mechanisms operate in the sea urchin embryo, we propose that overexpressed GID binds to endogenous GSK-3β and prevents it from interacting with Axin, thereby removing it from the endogenous destruction complex. Future work will examine this possibility in the sea urchin embryo.

## MATERIALS AND METHODS

### Care of animals and embryo culture

Adult *Strongylocentrotus purpuratus* were obtained from Marinus Scientific (Garden Grove, CA, USA) or from Pt Loma Marine Company (San Diego, CA, USA). Embryos were cultured in artificial sea water (ASW) at 15°C. Adult *Lytechinus variegatus* were obtained from Duke University Marine Lab (Beaufort, NC, USA) or Pelagic (Sugarloaf Key, FL, USA). Embryos were cultured in ASW at 25°C. Spawning was induced by intracoelomic injection of 0.5 M KCl.

### SpAxin cloning for mRNA expression

Full-length *S. purpuratus Axin* (*SpAxin*) cDNA was PCR-amplified from cDNA made from RNA collected from the egg stage using primers designed against the *SpAxin* sequence (Echinobase, SPU_001072). The primer sequences were: SpAxin forward primer, CGCGCGAATTCATGAGTCTAGAAGTGTATAG; SpAxin reverse primer, CGACCAGGCCTTGAGTGATCATCGACAGATTC. The PCR procedures followed the protocol for using Q5 high-fidelity DNA polymerase (NEB). The full-length SpAxin cDNA served as a template to make single domain deletions of Axin to remove the conserved APC, GSK-3β, Dvl and β-catenin-binding sites using standard molecular biology approaches. All clones were sequenced to validate the fidelity of the PCR protocol.

### Whole-mount *in situ* hybridization

The expression pattern of *SpAxin* in eggs and early embryos was determined using whole-mount *in situ* hybridization (WMISH). The *SpAxin in situ* hybridization probe was generated using a pair of primers specifically targeting *SpAxin*: SpAxin01-F, TAATACGACTCACTATAGGGAGCGTCAAGAGTGGTAAGC; SpAxin01-R, AATTAACCCTCACTAAAGGGTCGGTTGGAGGTAG. To perform WMISH, *S. purpuratus* eggs and embryos were fixed in a mixture of 4% (w/v) paraformaldehyde, 32.5 mM MOPS (pH 7.0), and 162.5 mM NaCl in filtered ASW at 4°C overnight. The WMISH protocol using digoxigenin-11-UTP-lablelled RNA probes was carried out as previously described (Bince et al., 2008). In each experiment, a final probe concentration of 0.1 ng/µl was used and the probe was detected using an alkaline phosphatase-conjugated anti-digoxigenin antibody (1:1500; Roche) and NBT/BCIP (Roche, Basel).

The effects of modulating Axin expression on endomesoderm gene expression was assayed using WMISH. Riboprobes for *L. variegatus* endomesoderm markers *Blimp1*, *Hox11/13b*, *Brachyury*, *FoxA*, *Delta* and *Gcm* were generated from linearized plasmids. Plasmids containing the *L. variegatus* cDNAs were generously provided by David McClay (Duke University, NC, USA) and Christine Byrum (College of Charleston, SC, USA). To detect endomesoderm gene expression, *L. variegatus* hatched blastula stage embryos were fixed in 8% paraformaldehyde (ThermoFisher Scientific) and 20 mM 4-(2-Hydroxyethyl)-1-piperazinepropanesulfonic acid (EPPS) in filtered ASW at 4°C overnight and the WMISH protocol was carried out as previously described in Byrum et al. (2009).

### Indirect immunofluorescence assays

For indirect immunofluorescence assays, embryos were stained as previously described (Peng and Wikramanayake, 2013). The embryos were fixed in 4% paraformaldehyde in phosphate-buffered saline (PBS, pH 7.4) at 4°C overnight. The mouse anti-Endo1 monoclonal antibody (1:20) was used to detect endoderm (Wessel and McClay, 1985).

### Microinjection of morpholino anti-sense oligonucleotides and mRNAs

For knockdown experiments, morpholino antisense oligonucleotides to target *S. purpuratus* and *L. variegatus Axin* mRNA (SpAxin or LvAxin MO), and a standard control morpholino (control MO) to a random nucleotide sequence (5′-CCTCTTACCTCAGTTACAATTTATA-3′) were obtained from Gene Tools. The SpAxin MO (5′-TATACACTTCTAGACTCATGATGGC-3′) and LvAxin MO (5'-ACCTATACACTTCCAAACTCATGGT-3′) were designed to span the start codon of the *Axin* transcripts (Fig. S1). The Axin MO or control MO was injected at a final concentration of 400 µM in 40% glycerol. For Axin overexpression experiments, the full-length *Axin* mRNA (*SpAxin*) and four *Axin* mRNAs, each with a single domain deletion (*SpAxinΔRGS*, *SpAxinΔGID*, *SpAxinΔβcat* and *SpAxinΔDAX*), along with the GID domain construct were synthesized. GFP mRNA was synthesized and used as a control. All Axin constructs were fused to GFP to facilitate detection of expression in the embryo. The full-length *Spβ-catenin* was synthesized (Genewiz) and fused with mCherry in the pCS2+ vector. The pCS2+ vector containing the respective cDNAs were linearized with NotI and mRNA was transcribed using the Ambion SP6 mMessage mMachine Kit (ThermoFisher Scientific). The mRNAs coding for the Axin constructs and Spβ-catenin::mCherry were mixed in 40% glycerol to a final concentration of 0.5 µg/µl and 0.2 µg/µl, respectively, and control *GFP* mRNA was injected at 0.109 µg/µl as previously described (Bince and Wikramanayake, 2008). For the GID domain experiments the *GID::GFP* mRNA and control *GFP* RNAs were injected at 0.5 µg/µl and 0.454 µg/µl, respectively. For microinjection, eggs were first fertilized in ASW containing 3-amino 1,2,4-triazole (ATA) to prevent the fertilization envelope from hardening and then injected immediately with a given MO or mRNA as previously described (Wikramanayake et al., 1998). The experiments were repeated at least three times. The survival rates of MO or mRNA injections were typically >90%. All the MO or mRNA injected embryos were imaged with a Zeiss Axiovert 200 inverted microscope. The effect of Axin MO or SpAxin overexpression on Spβ-catenin::mCherry nuclearization, was visualized using a Leica SP5 scanning confocal microscope.

### Quantitative PCR (qPCR)

To determine the effects of the injected Axin MO or the various Axin constructs on gene expression in the early embryo, quantitative PCR (qPCR) was performed using primers specific for genes expressed in endomesoderm and anterior neural ectoderm (ANE). qPCR was performed on *S. purpuratus* cDNA synthesized from total RNA extracted from morpholino- or mRNA-injected embryos. All injected embryos were collected at the hatching blastula stage (∼20 h post fertilization). Total RNA was isolated using the RNeasy Plus Micro Kit (Qiagen). The cDNA was synthesized from 50 ng of total RNA from control and experimental embryos using the qScript cDNA Synthesis kit (Quanta Biosciences). The PerfeCTa SYBR Green FastMix (Quanta Biosciences) was used for assembling the qPCR reactions. Each experiment was repeated at least three times with separate batches of embryos and each PCR reaction was carried out in triplicate. The expression of selected genes was analyzed using the delta delta Ct (2^-ΔΔCt^) method (Byrum et al., 2009; Range et al., 2013). With this method, Ct values of the targeted genes in both the MO- or mRNA-injected embryos were first adjusted to an internal control gene, *glyceraldehyde 3-phosphate dehydrogenase* (GAPDH, experimentally determined). For data analysis, the delta Ct was calculated for each sample as, for example, ΔCt_Axin-Mo_=Ct_GOI_-Ct_GAPDH_ and ΔCt_Control-Mo_=Ct_GOI_-Ct_GAPDH_. Then, the fold changes between Axin MO and control MO injected embryos were determined by 2^-ΔΔCt^, where ΔΔCt=ΔCt_Axin-Mo_-ΔCt_Control-Mo_. In qPCR analyses, the expression of *Blimp1*, *Hox11/13b*, *Endo 16, Brachyury, FoxA, GataE, Alx1*, *Delta*, *Gcm*, *Foxq2* and *Six3* were measured to evaluate the effects of injected MO or mRNA. For each gene, the log_2_ (fold change) values were used for statistical analyses using a two-tailed one-sample *t*-test against 1 (as no change in gene expression). All the primer sequences were downloaded from http://echinobase.org/Echinobase/q-pcr.html, but Foxq2 and Six3 primers were obtained from Range et al. (2013).

### Microsurgery

To determine whether Axin plays a direct role in downregulating cWnt signaling in anterior blastomeres, animal halves were isolated from Axin MO-injected *L. variegatus* embryos using a previously described protocol (Sweet et al., 2004; Wikramanayake et al., 1998). Briefly, this was achieved by injecting zygotes with the morpholinos and then using a baby eyelash to cut eight-cell stage embryos equatorially, and collecting the animal halves at the next cleavage stage as previously described (Sweet et al., 2004; Wikramanayake et al., 1998). Animal halves were collected from control MO-injected embryos as a control. For each experiment, 15-20 animal halves were obtained for morphological analysis and immunofluorescence assay for expression of Endo1, an endoderm gene marker (Wessel and McClay, 1985).

## Supplementary Material

Supplementary information

Reviewer comments
