# Supplementary material for: An early global role for Axin is required for correct patterning of the anterior-posterior axis in the sea urchin embryo
Source: Development. 2021 Mar 31;148(7):dev191197. doi: 10.1242/dev.191197 (PMC8034878; doi:10.1242/dev.191197)
Supplement: Supplementary information [file develop-148-191197-s1.pdf]

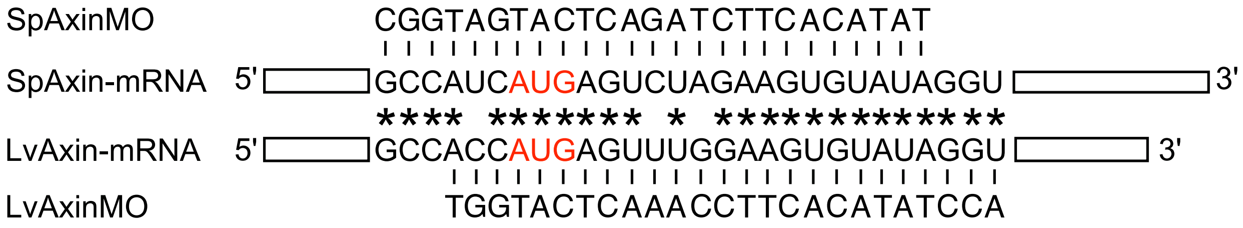

Figure S1- The design of sea urchin Axin Morpholino (MO) antisense oligonucleotides. Either *S. purpuratus* SpAxinMO or *L. variegatus* LvAxinMO was designed to span the start codon AUG (red) of *SpAxin* or *LvAxin* mRNA sequence respectively. Asterisks indicate the conserved nucleotides between SpAxin and LvAxin.

Development • Supplementary information

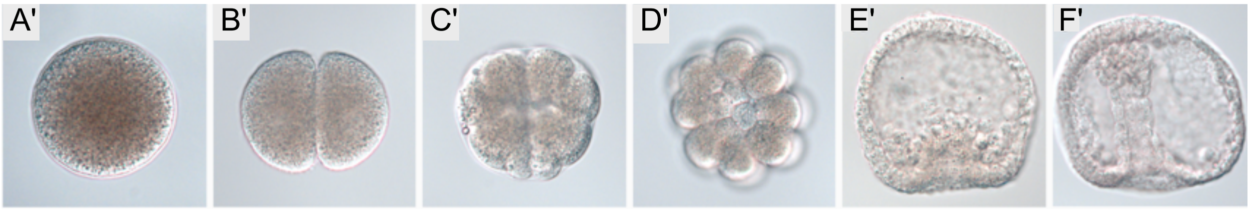

Figure S2- No signal was detected in WMISH when *S. purpuratus* eggs and embryos were probed with sense *Axin* probe. **(A)** egg; **(B)** 2-cell stage; **(C)** 16-cell stage; **(D)** 32-cell stage (the vegetal view of embryo); **(E)** early gastrula embryos; **(F)** late gastrula embryos.

Development • Supplementary information

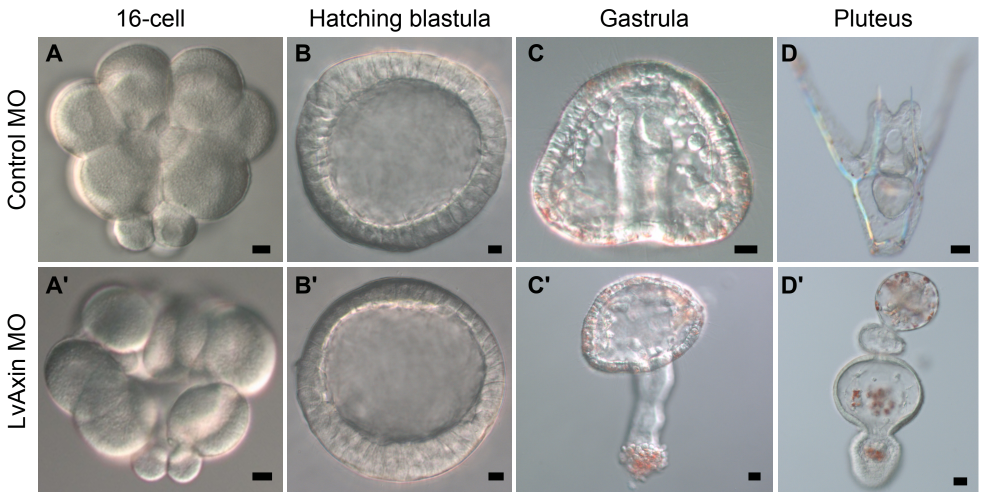

Figure S3- The effects on Axin knockdown on *L. variegatus* development. Axin MO was injected to zygotes to knockdown Axin protein expression. The standard Genetools control MO was injected as a negative control. At the early stages, from 16-cell to hatching blastula stage, there is no difference in morphology between embryos injected with Control MO **(A, B)** and embryos injected with Axin MO **(A', B')**. When controls were at gastrula **(C)** and pluteus **(D)** stages the Axin-knockdown embryos showed excess endomesoderm tissues and a posteriorized phenotype **(C', D')**. Scale bar = 10 μm. The concentrations for morpholino injections was 400 μM.

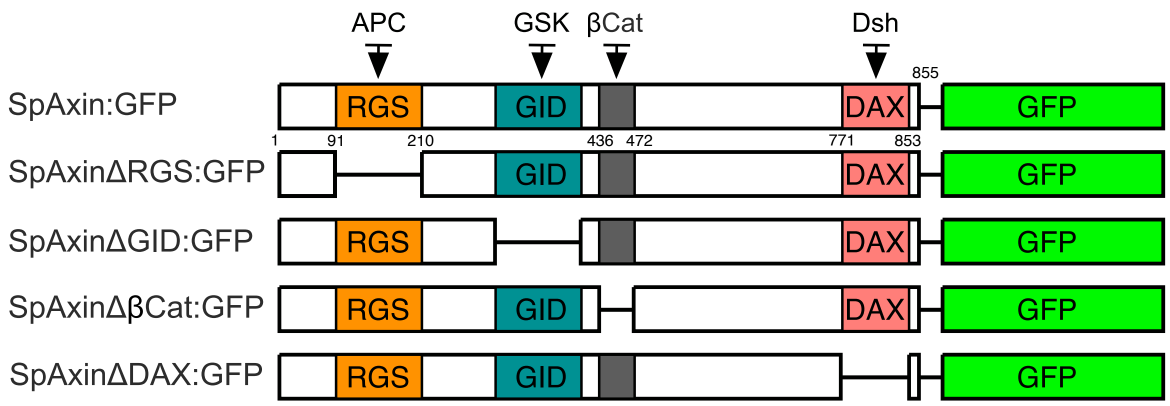

Figure S4- Schematic representation of Axin and mutant Axin constructs. All Axin constructs were fused to GFP.

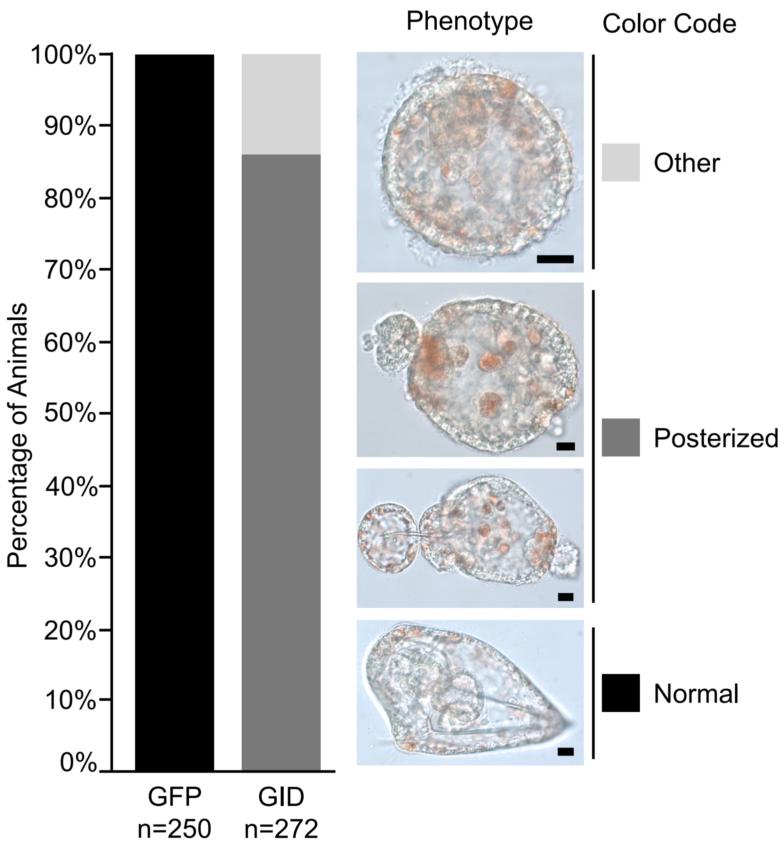

Figure S5- Quantification of the posteriorized phenotype induced in sea urchin embryos by overexpression of Axin GID::GFP. Left panel, bar graph shows the percentage of the different morphologies seen in GFP and Axin GID::GFP overexpressing embryos. Right panel, the morphology of the different phenotypes seen in the experiment. Color code corresponds to the colors of the bars. Scale bar = 10 µm. Experiments were done in *S. purpuratus*.

Development • Supplementary information
