# Supplementary material for: An early global role for Axin is required for correct patterning of the anterior-posterior axis in the sea urchin embryo
Source: Development. 2021 Mar 31;148(7):dev191197. doi: 10.1242/dev.191197 (PMC8034878; doi:10.1242/dev.191197)
Supplement: Reviewer comments [file develop-148-191197_review_history.pdf]

An early global role for Axin is required for correct patterning of the anterior-posterior axis in the sea urchin embryo

Hongyan Sun, Chieh-fu Jeff Peng, Lingyu Wang, Honglin Feng and Athula H. Wikramanayake
DOI: 10.1242/dev.191197

Editor: Cassandra Extavour

Review timeline

Original submission:	6 April 2020
Editorial decision:	29 May 2020
First revision received:	1 February 2021
Accepted:	25 February 2021

Original submission

First decision letter

MS ID#: DEVELOP/2020/191197

MS TITLE: A dual role for Axin in establishing the anterior-posterior axis in the early sea urchin embryo

AUTHORS: Hongyan Sun, Chieh-fu Jeff Peng, Lingyu Wang, Honglin Feng, and Athula H Wikramanayake

I have now received all the referees' reports on the above manuscript, and have reached a decision. The referees' comments are appended below, or you can access them online: please go to BenchPress and click on the 'Manuscripts with Decisions' queue in the Author Area.

As you will see, the referees express considerable interest in your work, but have some significant criticisms and recommend a substantial revision of your manuscript before we can consider publication. If you are able to revise the manuscript along the lines suggested, which may involve further experiments, I will be happy receive a revised version of the manuscript. Your revised paper will be re-reviewed by one or more of the original referees, and acceptance of your manuscript will depend on your addressing satisfactorily the reviewers' major concerns. Please also note that Development will normally permit only one round of major revision.

In my view, it will be particularly important to make it very clear in what ways your results provide new or unanticipated insights into mechanisms of Development. As you will see, a primary concern of Reviewer 1 is that these results were more or less expected based on studies from other organisms, so it will be critical to specify in what ways studying this well-known pathway in this sea urchin, tell us something about development that adds something new to our understanding.

We are aware that you may currently be unable to access the lab to undertake experimental revisions. If it would be helpful, we encourage you to contact us to discuss your revision in greater detail. Please send us a point-by-point response indicating where you are able to address concerns raised (either experimentally or by changes to the text) and where you will not be able to do so within the normal timeframe of a revision. We will then provide further guidance. Please also note that we are happy to extend revision timeframes as necessary.

© 2021. Published by The Company of Biologists under the terms of the Creative Commons Attribution License (https://creativecommons.org/licenses/by/4.0/).

Please attend to all of the reviewers' comments and ensure that you clearly highlight all changes made in the revised manuscript. Please avoid using 'Tracked changes' in Word files as these are lost in PDF conversion. I should be grateful if you would also provide a point-by-point response detailing how you have dealt with the points raised by the reviewers in the 'Response to Reviewers' box. If you do not agree with any of their criticisms or suggestions please explain clearly why this is so.

Reviewer 1

Advance summary and potential significance to field

Sun et al. provide a very careful and comprehensive study of the function of Axin in axis formation of sea urchin embryos. First, they find ubiquitous expression in cleavage stages and a vegetal expression later. By morpholino knock-down, they show that axin is required in animal blastomeres for repressing the canonical Wnt pathway (i.e. nuclear b-catenin) thereby contributing to the establishment of the a-p axis. Accordingly, MO animals were severely posteriorized on the level of both morphology and gene expression (checked by qPCR and WMISH). By separating treated animals into animal and vegetal halves the authors show that this effect is cell autonomous in the animal cells. Further, they confirm that this likely happens via the classical role of Axin as part of the b-catenin destruction complex as they could visualize loss of nuclear b-catenin in overexpression situation. Finally, they perform a comprehensive structure function analysis asking for the function of the conserved domains. They do so by overexpressing a series of deletion constructs both, in wt and axin knock-down. The GID domain, which is important for the binding of GSK to the destruction complex was found to be essential – in line with this, the overexpression of only the GID domain acted as a dominant negative. An interesting phenotype was found with the RGS deletion construct.

In summary, the authors provide the first comprehensive analysis of the function of axin in sea urchin (to my knowledge only misexpression studies of axin had been done before but with much less detailed analyses). Essentially, they find that Axin acts in the Wnt pathway in sea urchin in a similar way than in other species (i.e. probably being part of the destruction complex) and the phenotypes are by and large in line with the well described role of the Wnt pathway in axis formation of sea urchin and other animals. The structure-function analysis revealed results similar to those in other species for most domains but also an intriguing difference with respect to the RGS domain. On one hand, rescue experiments indicated that the RGS deletion construct was able to complement full length axin arguing for a non-essential role in this process. However, in the overexpression situation, the construct interfered with morphogenesis during gastrulation while it did not interfere with Wnt mediated gene regulation. The authors speculate on a function in morphogenesis independent of canonical Wnt signaling but did not follow this up in more detail.

The conclusions are backed by the data of this comprehensive and well-written manuscript. However, I found that its contribution of unexpected and novel insights to our understanding of developmental mechanisms remains somewhat limited as most of the findings are similar to those in other species and well within the current paradigm.

Comments for the author

Main suggestions for changes:
Discussion/Focus of the paper I was not completely convinced by the argument that in sea urchin the role of axin in axis formation goes beyond its canonical role in Wnt signaling. The authors find that axin is ubiquitously expressed at early stages – hence it does not appear to provide initial information on the a-p axis – it is “just” a component required for Wnt signaling in all cells. The signal initiating axis formation in sea urchins appears to be the asymmetrically distributed dishevelled (see Weitzel et al. 2004 and others). I understand that in beetles, Axin itself appears to be the asymmetrically localized signal that initiates polarity. In essence, in sea urchins Axin appears to do its canonical job in Wnt signaling while other factors initiate the asymmetry. Therefore, I suggest reducing or removing the parts of the discussion, which deal with the evolution of axis formation.
The potentially novel role of the RTG domain is an important point of the discussion and might gain more visibility instead.

© 2021. Published by The Company of Biologists under the terms of the Creative Commons Attribution License (https://creativecommons.org/licenses/by/4.0/).

Vertebrates/invertebrates I feel that results should be discussed in the light of the accepted separation of animal phylogeny into deuterostomes and protostomes. Using the “old” distinction between vertebrates and invertebrates might obscure relationships.

Results:
In the overexpression of the deletion constructs: Some experiments appear to show an increased gene overexpression compared to the wt construct. Is that difference significant enough to follow it up? Could these results indicate a role of these domains in negative feedback regulation of the Wnt pathway? Might be interesting.

Typos etc:
Gene name convention In many species, the protein is given in regular font (often upper case), while the gene is italic (often in lower case). Please adjust according to the convention in your field for axin and catenin and others.
Sometimes, you add a species prefix (Spaxin) and sometimes not. I find it a good idea to add the suffix – please consider separating with a dash and using a 3-letter suffix “Spu-axin”)
Introduction:
p.4 The AP axis is not usually the first to be established – often, both are established at the same time.
Refs are missing for the statement that Wnt signaling is involved in axis formation in many animals (end of first paragraph).
p.6 proteasome p.9 In Fig. 1 please label the structures indicated in the text (like micromeres etc). For a non-sea-urchinologist a little scheme with axes and blastomere naming would have helped. please explain “veg2 tier” at first use

Results:
p.11 I was a bit confused with the two species here. Options: First describe the results from your main species (shown in the main fig) and only then refer to S3, i.e. the other species in the supplementary. Or mention that the analysis was done in both species right at the beginning of the paragraph.
Is it convention to write “Control embryos” in upper case?
P 14
…Range, 2014, 2018;) (space and ; too much – or citation missing)
P16
“were all able to”
Please mark oral hood in the panel
“with no obvious morphological differences” – to my eyes, the embryo in S3 A’ looked quite deranged compared to the wt one.
P17
“The intact anterior-posterior polarity indicated that…” (right?)
You ascribe the cWnt independent function to non-canoncial Want. That is possible – but could it also be something completely different? If so then stay cautious here by just stating “b-catenin independent function”

Discussion:
p. 20 From “Mechanistically, this anteriorization…” to “ANE territory (Range et al. 2013).” There is some redundancy,
p.21 suggestion for alternative phrasing “…failed to posteriorize embryos as a dominant-negative to endogenous Axin would do.”
P22
…morphogenetic event such as apical…
P23 Maybe a for paragraph on the GID domain?
p.24
“to the best of our knowledge a role for Axin in suppressing cWnt…not been demonstrated except in T. cast…”
As mentioined above I have difficulties with this statement – in all species, repression of cWnt always is supposed to rely on the destruction complex involving Axin. Hence, this general role is self-evident. A specific function in initiating the signal was not shown for sea urchin.

© 2021. Published by The Company of Biologists under the terms of the Creative Commons Attribution License (https://creativecommons.org/licenses/by/4.0/).

p. 24/25 This discussion on the evolution of the animal-vegetal axis is not enriched by the data provided in the paper. Hence, the paragraph could be omitted. And on a personal note: I am not convinced of the animal-vegetal flip of Wnt activity – and I know not many colleagues who are.

Reviewer 2

Advance summary and potential significance to field

In this manuscript entitled “A dual role for Axin in establishing the anterior-posterior axis in the early sea urchin embryo” the authors report their findings showing how Axin plays an essential role within the cWnt pathway that is necessary for specification and patterning the early sea urchin embryo along the anterior-posterior axis. They use morpholino and dominant negative perturbation studies to clearly show that Axin is an critical component of the “destruction complex” that degrades cytoplasmic beta-catenin in the absence of Wnt signaling. The most significant findings the structure-function analyses that shows that the GSK3-beta binding site in Axin is the most critical protein interaction domain, something that has been not clearly understood in other vertebrate and Drosophila studies. Together, these experiments move the field forward and provide further evidence for the deep homology of the cWnt pathway among animals. In addition the manuscript further establishes the sea urchin as an important in vivo developmental model system for understanding the intricacies of cell-to-cell signaling and their evolution.

Comments for the author

The the overall study is well-designed and executed and I do not believe they need many further experiments. However, I think the manuscript contains some issues that should be addressed before consideration for acceptance. My comments are listed below.

Major issues:

1) I think the authors overstate the localized role of Axin in the anterior cells of the embryo. The role of the destruction complex, which includes Axin, is to degrade cytoplasmic beta-catenin in every cell of the the embryo preventing it from entering the nucleus and activating gene transcription. Thus, one would expect Axin to be expressed in most, if not all, cells where it prevents ectopic cWnt activity. The authors have previously shown that membrane localized Disheveled is necessary in the posterior pole of the embryo to override the function of the Axin/destruction complex, allowing cWnt signaling where Disheveled is membrane localized. This issue should be addressed in the main body and the discussion.

2) Related to issue 1, the authors show that axin mRNA is up regulated in posterior endoderm and mesoderm cells compared to other cells at the beginning of gastrulation. This finding is interesting. Does this mean that more Axin is necessary for gastrulation/specification of these cells? The authors do not significantly address this finding in the results and/or discussion sections.

3) The authors show that by over expressing the the delta-RGS-Axin mRNA construct that gastrulation is perturbed, but that neither endomesoderm nor anterior GRN components are affected. To me this suggests that the Axin + APC interaction is both essential for cWnt signaling as well as gastrulation. The authors touch on this idea, but neglect to discuss the possibility that Axin could be involved in the non-canonical Wnt signaling pathways that have been shown to be necessary for gastrulation in the sea urchin embryo. At least one paper has shown that Axin is involved as a component in both cWnt and non-canonical pathways within the same cells (see Grumalato et al. Genes and Development 2010). The authors should consider discussing this possibility in the main body and the discussion. In addition, they might consider in situ hybridization assays to determine if these embryos are anteriorized.

4) I might have missed it, but the S. purpuratus Axin MO phenotype did not appear clearly in the paper.

Minor issues:

© 2021. Published by The Company of Biologists under the terms of the Creative Commons Attribution License (https://creativecommons.org/licenses/by/4.0/).

Figure 1 - the authors show that axin mRNA is up regulated in posterior cells. Do the authors believe that axin is completely down regulated in anterior cells in the blastula/gastrula stages. This would be a surprising result given the essential nature of the ubiquitous destruction complex? Also, the authors should consider labeling the AP axis in the diagram.

Figure 2 - to help the reader the authors should consider labeling the AP axis on the diagram.

Figure 2 and 5 - Since there are 2 morpholinos used in 2 different sea urchin embryos, it would be helpful to be clear in the figure images which species is being represented.

Figure 8 - The delta-RGS figure does not look like a completely anteriorized embryo. This should be discussed.

First revision

Author response to reviewers' comments

We thank both reviewers for the thoughtful reviews on the manuscript and we have responded to all of their comments/concerns. The point-by-point responses are shown below. We have also made major edits to the abstract and the rest of the manuscript to ensure that we are adhering to the word limits for the journal. Where we have made the changes suggested by the reviewers, we have indicated the page number to make it easy for the reviewers to see the changes. We have also changed the title of the manuscript to “An early global role for Axin is required for correct patterning of the anterior-posterior axis in the sea urchin embryo” because it more accurately reflects the focus of the revised manuscript. Please note that we have changed the discussion significantly to emphasize the novel insights provided by this work. Our responses to the individual reviewer comments are shown in blue text below (please see PDF version of these responses in the supplemental documents).

Responses to Reviewer comments:

Reviewer 1 Advance summary and potential significance to field
Sun et al. provide a very careful and comprehensive study of the function of Axin in axis formation of sea urchin embryos. First, they find ubiquitous expression in cleavage stages and a vegetal expression later. By morpholino knock-down, they show that axin is required in animal blastomeres for repressing the canonical Wnt pathway (i.e. nuclear b-catenin) thereby contributing to the establishment of the a-p axis. Accordingly, MO animals were severely posteriorized on the level of both morphology and gene expression (checked by qPCR and WMISH). By separating treated animals into animal and vegetal halves the authors show that this effect is cell autonomous in the animal cells. Further, they confirm that this likely happens via the classical role of Axin as part of the b-catenin destruction complex as they could visualize loss of nuclear b-catenin in overexpression situation. Finally, they perform a comprehensive structure function analysis asking for the function of the conserved domains. They do so by overexpressing a series of deletion constructs both, in wt and axin knock-down. The GID domain, which is important for the binding of GSK to the destruction complex was found to be essential – in line with this, the overexpression of only the GID domain acted as a dominant negative. An interesting phenotype was found with the RGS deletion construct.

In summary, the authors provide the first comprehensive analysis of the function of axin in sea urchin (to my knowledge only misexpression studies of axin had been done before but with much less detailed analyses). Essentially, they find that Axin acts in the Wnt pathway in sea urchin in a similar way than in other species (i.e. probably being part of the destruction complex) and the phenotypes are by and large in line with the well described role of the Wnt pathway in axis formation of sea urchin and other animals. The structure-function analysis revealed results similar to those in other species for most domains but also an intriguing difference with respect to the RGS domain. On one hand, rescue experiments indicated that the RGS deletion construct was able to

© 2021. Published by The Company of Biologists under the terms of the Creative Commons Attribution License (https://creativecommons.org/licenses/by/4.0/).

complement full length axin arguing for a non-essential role in this process. However, in the overexpression situation, the construct interfered with morphogenesis during gastrulation while it did not interfere with Wnt mediated gene regulation. The authors speculate on a function in morphogenesis independent of canonical Wnt signaling but did not follow this up in more detail.

The conclusions are backed by the data of this comprehensive and well-written manuscript. However, I found that its contribution of unexpected and novel insights to our understanding of developmental mechanisms remains somewhat limited as most of the findings are similar to those in other species and well within the current paradigm.

Reviewer 1 Comments for the author
Main suggestions for changes:

Discussion/Focus of the paper
1) I was not completely convinced by the argument that in sea urchin the role of axin in axis formation goes beyond its canonical role in Wnt signaling. The authors find that axin is ubiquitously expressed at early stages – hence it does not appear to provide initial information on the a-p axis – it is “just” a component required for Wnt signaling in all cells. The signal initiating axis formation in sea urchins appears to be the asymmetrically distributed dishevelled (see Weitzel et al. 2004 and others). I understand that in beetles, Axin itself appears to be the asymmetrically localized signal that initiates polarity. In essence, in sea urchins Axin appears to do its canonical job in Wnt signaling while other factors initiate the asymmetry. Therefore, I suggest reducing or removing the parts of the discussion, which deal with the evolution of axis formation.

We have now revised the discussion significantly to emphasize how this work provides novel insight that we believe is broadly relevant to the correct AP patterning in other taxa also. It is true that our work shows that Axin is functioning in its expected role in the beta-catenin destruction complex, but to the best of our knowledge, no other studies have highlighted the critical need to have an active destruction complex in all blastomeres in rapidly dividing embryos to tightly regulate the fluctuation of beta-catenin with the cell cycle. The phenotypes that we see for Axin knockdown and those that have been reported for Axin knockout/knockdown in other deuterostomes indicates that in the absence of this protein there is a duplication of posterior/dorsal structures in embryos.

As suggested by Reviewer 1, we have removed the part of the discussion that deals with the evolution of axis formation

2) The potentially novel role of the RTG domain is an important point of the discussion and might gain more visibility instead.

We agree that this is an interesting observation that we will be following up on. We have provided an extensive discussion on the possible underlying mechanisms for the phenotypes seen in our experiment when we use the Axin ΔRGS construct (pg. 23/24).

3)Vertebrates/invertebrates
I feel that results should be discussed in the light of the accepted separation of animal phylogeny into deuterostomes and protostomes. Using the “old” distinction between vertebrates and invertebrates might obscure relationships.

We have now changed our use of the terms as suggested, where it is appropriate in the text.

4) Results:
In the overexpression of the deletion constructs: Some experiments appear to show an increased gene overexpression compared to the wt construct. Is that difference significant enough to follow it up? Could these results indicate a role of these domains in negative feedback regulation of the Wnt pathway? Might be interesting.

We agree that they are intriguing results that we are following up on. But we feel that these results would be beyond the scope of the current work in this manuscript.

© 2021. Published by The Company of Biologists under the terms of the Creative Commons Attribution License (https://creativecommons.org/licenses/by/4.0/).

Typos etc:

5) Gene name convention
In many species, the protein is given in regular font (often upper case), while the gene is italic (often in lower case). Please adjust according to the convention in your field for axin and catenin and others.
Sometimes, you add a species prefix (Spaxin) and sometimes not. I find it a good idea to add the suffix – please consider separating with a dash and using a 3-letter suffix "Spu-axin")

We have made adjustments to make sure that all gene names conform to what is general practice in the field.

6) Introduction:
p.4
The AP axis is not usually the first to be established – often, both are established at the same time.

We respectfully disagree with the reviewer on this point. There are several influential references that indicate that the AP axis is the axis that is specified first in most animal taxa (Please see Kume and Dan, 1968; Wilson, 1925 in the reference list and Goldstein and Freeman, 1996, BioEssays, 19, 105-116).

7) Refs are missing for the statement that Wnt signaling is involved in axis formation in many animals (end of first paragraph).

This has been added

8) p.6
proteasome

This has been corrected (pg. 5).

9) p.9
In Fig. 1 please label the structures indicated in the text (like micromeres etc). For a non-sea-urchinologist a little scheme with axes and blastomere naming would have helped.
please explain "veg2 tier" at first use

We have now added the labels as requested (see Fig 1,3,4 and 5 for example), we have indicated the orientation of the axes of the embryos in the figures and have explained the position of the "veg2 tier" (Pg. 9, line 1).

9) Results:
p.11
I was a bit confused with the two species here. Options: First describe the results from your main species (shown in the main fig) and only then refer to S3, i.e. the other species in the supplementary. Or mention that the analysis was done in both species right at the beginning of the paragraph.

As suggested by Reviewer 1, we have mentioned that the analyses were done on both species at the start of the results section (Pg. 8). In addition, each figure legend clearly indicates the species that the data was generated from.

10) Is it convention to write "Control embryos" in upper case?

We are not aware if there is a conventional way to do this. But we have now switched to lower case.

11) P 14
…Range, 2014, 2018;) (space and ; too much – or citation missing)

© 2021. Published by The Company of Biologists under the terms of the Creative Commons Attribution License (https://creativecommons.org/licenses/by/4.0/).

This has been fixed.

12) P16
"were all able to"

This has been corrected.
13) Please mark oral hood in the panel

We have now added arrowheads to mark the oral hoods in Figure 7.

14) "with no obvious morphological differences" – to my eyes, the embryo in S3 A' looked quite deranged compared to the wt one.

This embryo is actually normal in its development. We think that it looks a little different from the control embryos because of the plane of focus for the image capture and also because some cells may be starting to initiate mitosis.

14) P17
"The intact anterior-posterior polarity indicated that…" (right?)

We have edited this sentence.

15) You ascribe the cWnt independent function to non-canoncial Want. That is possible – but could it also be something completely different? If so then stay cautious here by just stating "b-catenin independent function"

Yes, we agree. This has been changed (Pg 16)

Discussion:
15) p. 20
From "Mechanistically, this anteriorization…" to "ANE territory (Range et al. 2013)." There is some redundancy,

We have edited the paragraph to remove the redundancy (Pg 22)

16) p.21
suggestion for alternative phrasing "…failed to posteriorize embryos as a dominant-negative to endogenous Axin would do."

We have made the changes as suggested by Reviewer 1 (Pg 23).

17) P22
…morphogenetic event such as apical…

We have made the edit (Pg. 24).

18) P23
Maybe a for paragraph on the GID domain?

We discuss GID in a separate paragraph (Pg. 25)

19) p.24
"to the best of our knowledge a role for Axin in suppressing cWnt…not been demonstrated except in T. cast…"

As mentioined above I have difficulties with this statement – in all species, repression of cWnt always is supposed to rely on the destruction complex involving Axin. Hence, this general role is self-evident. A specific function in initiating the signal was not shown for sea urchin.

© 2021. Published by The Company of Biologists under the terms of the Creative Commons Attribution License (https://creativecommons.org/licenses/by/4.0/).

We have now removed this line and have emphasized the developmental significance of our findings in the first section of the revised discussion (Pg.19-21).

20) p. 24/25
This discussion on the evolution of the animal-vegetal axis is not enriched by the data provided in the paper. Hence, the paragraph could be omitted. And on a personal note: I am not convinced of the animal-vegetal flip of Wnt activity – and I know not many colleagues who are.

We have removed the mentioned paragraphs from the revised manuscript.

Reviewer 2 Advance summary and potential significance to field

In this manuscript entitled “A dual role for Axin in establishing the anterior-posterior axis in the early sea urchin embryo” the authors report their findings showing how Axin plays an essential role within the cWnt pathway that is necessary for specification and patterning the early sea urchin embryo along the anterior-posterior axis. They use morpholino and dominant negative perturbation studies to clearly show that Axin is an critical component of the “destruction complex” that degrades cytoplasmic beta-catenin in the absence of Wnt signaling. The most significant findings the structure-function analyses that shows that the GSK3-beta binding site in Axin is the most critical protein interaction domain, something that has been not clearly understood in other vertebrate and Drosophila studies. Together, these experiments move the field forward and provide further evidence for the deep homology of the cWnt pathway among animals. In addition the manuscript further establishes the sea urchin as an important in vivo developmental model system for understanding the intricacies of cell-to-cell signaling and their evolution.

Reviewer 2 Comments for the author
The the overall study is well-designed and executed and I do not believe they need many further experiments. However, I think the manuscript contains some issues that should be addressed before consideration for acceptance. My comments are listed below.

Major issues:

1) I think the authors overstate the localized role of Axin in the anterior cells of the embryo. The role of the destruction complex, which includes Axin, is to degrade cytoplasmic beta-catenin in every cell of the the embryo preventing it from entering the nucleus and activating gene transcription. Thus, one would expect Axin to be expressed in most, if not all, cells where it prevents ectopic cWnt activity. The authors have previously shown that membrane localized Disheveled is necessary in the posterior pole of the embryo to override the function of the Axin/destruction complex, allowing cWnt signaling where Disheveled is membrane localized. This issue should be addressed in the main body and the discussion.

We have now addressed this issue in the discussion. It is correct that you would expect to see activity of the destruction complex in all or most cells. But as we argue in the discussion, the levels of Axin in the egg and early embryo are much higher compared to Axin expression (outside the vegetal plate) at the late blastula stage and beyond. This is also true in other deuterostomes where Axin expression in eggs and early embryos has been documented. We suggest that high levels of Axin are needed in early rapidly cleaving blastomeres to protect these cells with broad potential from assuming inappropriate cell fates. This would be critical spatially, but also temporally. Nuclear beta-catenin is not seen in the embryo until the 16-cell stage, and the destruction complex may tightly regulate the cytoplasmic levels in all cells, until the Dishevelled-mediated mechanism activates cWnt signaling in posterior blastomeres. As we have outlined in the discussion, this mechanism may be a general phenomenon in other deuterostome embryos as well. As such, we believe that this is an important heretofore under-appreciated aspect of early patterning in metazoan embryos.

2) Related to issue 1, the authors show that axin mRNA is up regulated in posterior endoderm and mesoderm cells compared to other cells at the beginning of gastrulation. This finding is interesting.

© 2021. Published by The Company of Biologists under the terms of the Creative Commons Attribution License (https://creativecommons.org/licenses/by/4.0/).

Does this mean that more Axin is necessary for gastrulation/specification of these cells? The authors do not significantly address this finding in the results and/or discussion sections.

We have discussed this in the revised document (Pg. 20).

3) The authors show that by over expressing the the delta-RGS-Axin mRNA construct that gastrulation is perturbed, but that neither endomesoderm nor anterior GRN components are affected. To me this suggests that the Axin + APC interaction is both essential for cWnt signaling as well as gastrulation. The authors touch on this idea, but neglect to discuss the possibility that Axin could be involved in the non-canonical Wnt signaling pathways that have been shown to be necessary for gastrulation in the sea urchin embryo. At least one paper has shown that Axin is involved as a component in both cWnt and non-canonical pathways within the same cells (see Grumalato et al. Genes and Development 2010). The authors should consider discussing this possibility in the main body and the discussion. In addition, they might consider in situ hybridization assays to determine if these embryos are anteriorized.

We discuss this in pg. 23/24. As we have indicated in the manuscript, these embryos are not anteriorized because they show relatively normal endomesodermal gene expression.

4) I might have missed it, but the S. purpuratus Axin MO phenotype did not appear clearly in the paper.

The S. purpuratus MO phenotype is shown in Figure 3. We have now clearly indicated the species that the data was generated from in the figure legends.

Minor issues:

Figure 1 - the authors show that axin mRNA is up regulated in posterior cells. Do the authors believe that axin is completely down regulated in anterior cells in the blastula/gastrula stages. This would be a surprising result given the essential nature of the ubiquitous destruction complex? Also, the authors should consider labeling the AP axis in the diagram.

This is a good point that the reviewer brings up. By WMISH there appears to be low levels of expression in the anterior blastomeres. We would have to do measurements in isolated animal and vegetal halves to quantify the differences using qPCR. This is technically feasible, but logistically not possible since the lead author who is an expert on this method has graduated.

We have labeled the AP axis in images where necessary.

Figure 2 - to help the reader the authors should consider labeling the AP axis on the diagram.

We have done this (see Fig 3,4 and 5)

Figure 2 and 5 - Since there are 2 morpholinos used in 2 different sea urchin embryos, it would be helpful to be clear in the figure images which species is being represented.

We apologize for this oversight. The species names have been added to all the figures.

Figure 8 - The delta-RGS figure does not look like a completely anteriorized embryo. This should be discussed.

We agree with this observation. We have discussed this in the results. The delta-RGS construct strongly inhibits gastrulation but does not adversely affect gene expression. Hence, by morphology and gene expression it is not anteriorized.

We hope that we have addressed the reviewers concerns adequately and that with these changes you will find that the work has the significance to be published in Development.

© 2021. Published by The Company of Biologists under the terms of the Creative Commons Attribution License (https://creativecommons.org/licenses/by/4.0/).

Second decision letter

MS ID#: DEVELOP/2020/191197

MS TITLE: An early global role for Axin is required for correct patterning of the anterior-posterior axis in the sea urchin embryo

AUTHORS: Hongyan Sun, Chieh-fu Jeff Peng, Lingyu Wang, Honglin Feng, and Athula H Wikramanayake
ARTICLE TYPE: Research Article

I am happy to tell you that your manuscript has been accepted for publication in Development, pending our standard ethics checks.

Reviewer 1

Advance summary and potential significance to field

The authors provide the first comprehensive analysis of the function of Axin in sea urchin. Essentially, they find that Axin acts in the canonical Wnt pathway in sea urchin in a similar way than in other species (i.e. being part of the destruction complex) and the phenotypes are by and large in line with the previously described role of the Wnt pathway in axis formation of sea urchin and other animals. The structure-function analysis revealed similar functions for a number of domains. An intriguing role was found with respect to the RGS domain. The work thoroughly describes the function of Axin in early sea urchin embryos and presents interesting novel findings, which should inspire further research.

Comments for the author

The authors have implemented appropriate changes to the manuscript, which I now highly recommend for publication in this form.

Some minor points at the discretion of the authors:

Abstract:
- "… Deuterostome, we studied Axin function in …"

End of introduction:
- "…role in early AP axis patterning in early embryos" (two times early)

Discussion:
Suggestion to emphasize more:
"…divergent functions with the GSK-3β-binding region.."
"…divergent functions. Importantly, the GSK-3β-binding region…"

Suggestion to emphasize more would be starting the paragraph in a different way:
"Many in vitro studies done in cultured cells and in vivo studies done primarily in vertebrates and …"
"One of our most important findings in the structure function analysis was that ……. Many in vitro studies done in …."

Reviewer 2

Advance summary and potential significance to field

This revision is a significant improvement over the previous version. The writing, changes and re-organization of the manuscript makes it an easier story to follow. The authors addressed most of

© 2021. Published by The Company of Biologists under the terms of the Creative Commons Attribution License (https://creativecommons.org/licenses/by/4.0/).

my major criticisms and I believe it is a significant contribution to a poorly understood fundamental mechanism in most organisms.

I recommend this manuscript for publication.

Comments for the author

I do not encourage revision.

© 2021. Published by The Company of Biologists under the terms of the Creative Commons Attribution License (https://creativecommons.org/licenses/by/4.0/).
